# Automated Curation of CNMF-E-Extracted ROI Spatial Footprints and Calcium Traces Using Open-Source AutoML Tools

**DOI:** 10.3389/fncir.2020.00042

**Published:** 2020-07-15

**Authors:** Lina M. Tran, Andrew J. Mocle, Adam I. Ramsaran, Alexander D. Jacob, Paul W. Frankland, Sheena A. Josselyn

**Affiliations:** ^1^Hospital for Sick Children, Neurosciences and Mental Health, Toronto, ON, Canada; ^2^Department of Physiology, University of Toronto, Toronto, ON, Canada; ^3^Postgraduate Affiliates Program, Vector Institute, Toronto, ON, Canada; ^4^Department of Psychology, University of Toronto, Toronto, ON, Canada; ^5^Institute of Medical Sciences, University of Toronto, Toronto, ON, Canada; ^6^Child & Brain Development Program, Canadian Institute for Advanced Research (CIFAR), Toronto, ON, Canada; ^7^Brain, Mind & Consciousness Program, Canadian Institute for Advanced Research (CIFAR), Toronto, ON, Canada

**Keywords:** calcium imaging, open-source, machine learning, microendoscopy, 1-photon, CNMF-E

## Abstract

*In vivo* 1-photon (1p) calcium imaging is an increasingly prevalent method in behavioral neuroscience. Numerous analysis pipelines have been developed to improve the reliability and scalability of pre-processing and ROI extraction for these large calcium imaging datasets. Despite these advancements in pre-processing methods, manual curation of the extracted spatial footprints and calcium traces of neurons remains important for quality control. Here, we propose an additional semi-automated curation step for sorting spatial footprints and calcium traces from putative neurons extracted using the popular constrained non-negative matrixfactorization for microendoscopic data (CNMF-E) algorithm. We used the automated machine learning (AutoML) tools TPOT and AutoSklearn to generate classifiers to curate the extracted ROIs trained on a subset of human-labeled data. AutoSklearn produced the best performing classifier, achieving an F1 score >92% on the ground truth test dataset. This automated approach is a useful strategy for filtering ROIs with relatively few labeled data points and can be easily added to pre-existing pipelines currently using CNMF-E for ROI extraction.

## Introduction

Advances in 1-photon (1p) miniaturized fluorescence microscopy in terms of utility, cost, and ease-of-use have increased the accessibility and popularity of *in vivo* calcium imaging in freely behaving rodents (Ghosh et al., 2011; Hamel et al., 2015; Cai et al., 2016; Jacob et al., 2018). Consequently, researchers can track the activity of neuronal populations across days, weeks, or even months (Rubin et al., 2015; Gonzalez et al., 2019). Concurrent with the growing usage of 1p microendoscopy in neuroscience, there is an increasing demand for high-throughput software that can accurately and efficiently process the very large raw calcium imaging datasets now being produced. To address this challenge, several algorithms and analysis pipelines have been developed to automate the extraction of cells and calcium activity traces across time in a robust manner—necessary step for downstream analyses (Pnevmatikakis, 2019).

Motion correction, source extraction, and cell registration (across multiple recording sessions) are important steps involved in pre-processing raw 1p calcium imaging data. Source extraction, the task of identifying the locations and activity of neurons in the imaged field of view (FOV), is arguably the most challenging of these steps, as evidenced by the number of different algorithms published to improve this step. Nevertheless, two main methods of source extraction have been widely adopted in the field: principal component analysis/independent component analysis (PCA/ICA; Mukamel et al., 2009) and the more recent extended constrained non-negative matrix factorization for microendoscopic data (CNMF-E; Zhou et al., 2018). CNMF-E explicitly models background signals present in 1p microendoscopic recordings and therefore results in more accurate signal detection from neurons compared to PCA/ICA (Zhou et al., 2018).

Our lab has successfully applied CNMF-E to recordings from our open-source Compact Head-mounted Endoscope (CHEndoscope) to identify neuron locations (or spatial footprints) and extract their calcium activity traces from freely-behaving mice performing different behavioral tasks. CNMF-E has proven to be a reliable tool across multiple imaging sessions and experimental paradigms conducted in the lab with minimal parameter tuning in our hands (Jacob et al., 2018). However, like PCA/ICA, CNMF-E may still produce some false-positives in the output of detected cells (i.e., non-neuronal spatial footprints or calcium traces), which can be filtered out of the final dataset manually. We initially found success in filtering CNMF-E-extracted spatial footprints and traces by adding a manual curation step that involved visual inspection of each ROI and calcium trace (previously described in Jacob et al., 2018). While this type of manual curation can reduce the number of false-positives in CNMF-E’s output, visual inspection of potentially tens of thousands of extracted cells can be time-consuming, and this method is not free from human error. Here, we propose an automated machine learning (AutoML) approach built on top of the CNMF-E algorithm’s outputs to filter out potential false-positives. We implemented a semi-automated classification tool to limit the amount of manual curation required during pre-processing, without completely removing the ability to fine-tune the process with human-labeled datasets.

The main outputs of CNMF-E’s source extraction algorithm are: 1. the extracted calcium traces representing cellular activity and 2. the spatial footprint of putative neurons. As mentioned previously, manual curation of these outputs involves identifying both aberrant traces that do not have stable baseline fluorescence (Resendez et al., 2016), transient durations inconsistent with the expressed calcium indicator (e.g., GCAMP6f; Badura et al., 2014), and/or spatial footprints that are not consistent with the shape and size of neurons in the brain region being recorded (Resendez et al., 2016). We trained and validated our classifiers on a dataset of 14,000 manually curated spatial footprints and traces output from CNMF-E. The final model chosen was then used to automate the curation of ROIs from other recording sessions. From the two AutoML libraries, we chose the best performing model to train on the full training set to evaluate on the test set. We find our model can accurately predict whether a cell would be included or excluded at a rate of 92%. We further validated the performance of our models on an additional ground truth dataset derived from 2-photon (2p) imaging source ROIs of mouse visual cortex (Stringer et al., 2019a,b), where ROIs were downsampled to emulate 1p images and modified to simulate a proportion of “negative” labeled ROIs for ground truth labels. The AutoML classifier was able to accurately predict >98% of the correct labels on the 2p simulated ground-truth dataset.

The potential time savings of manually curating thousands of cells makes this approach a method worth employing as part of a typical 1p calcium imaging pipeline. While our AutoML-based curation pipeline was primarily developed to be used with CHEndoscope data, our model takes the output of CNMF-E and as a result, allows this method to be readily applied to data acquired using other 1p miniature microscopes.

## Materials and Methods

### Dataset Preparation and Pre-processing

The dataset used for model training was acquired from multiple hippocampal CA1 recordings captured across different mice and recording sessions using methods described in Jacob et al., 2018. From these recordings, we used CNMF-E (Zhou et al., 2018) to extract spatial footprints and calcium traces of 14,000 ROIs. We then manually reviewed and labeled these ROIs as neuronal (included for further analysis) or artifact (excluded from analysis). The labels were generated by two human expert raters that inspected the calcium transients and spatial footprints based on previously reported criteria:

1. Fast rise and slow decay of calcium transients with stable baseline fluorescence (Resendez et al., 2016).
2. Calcium transient durations consistent with GCaMP6f (or appropriate GCaMP variant; Badura et al., 2014).
3. Spatial footprints consistent with appropriate neuronal shape and size (Resendez et al., 2016).

**Figure 1:**
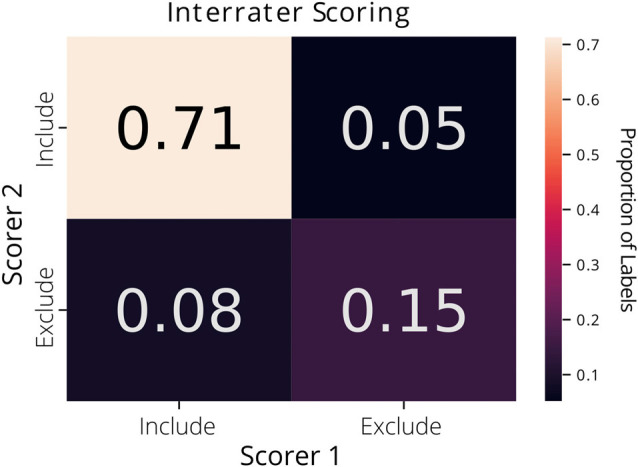
Interrater agreement of ROI labels. A confusion matrix comparing the manually reviewed labels (include or exclude) for putative ROIs extracted from constrained non-negative matrix factorization for microendoscopic data (CNMF-E) determined by two different raters. Each cell of the matrix is annotated with the proportion of ROIs.

Each label in the ground truth dataset was derived from a single rater’s annotations. Interrater agreement was 87% across the two raters on a subset of the data (1,073 putative ROIs extracted from CNMF-E; Figure 1).

Spatial footprints consisted of the maximum projection of the identified cell from all frames in the video. We found that the location of the footprint in the FOV was not important in our labeling criteria (compared to the shape and size of footprint), we cropped the spatial footprints to remove empty space. Each spatial footprint was reduced to an 80 × 80 pixel image centered on the peak intensity of the footprint. Furthermore, recordings were of varying lengths, so all trace data was cropped at 500 frames (equivalent to 100 s of recording at 5 fps) and normalized such that the values. To combine the spatial and trace data into a single dataset for classification, the 2-dimensional spatial footprint images were flattened and the trace data were concatenated to the end of the image data to create a single 1-dimensional vector for each ROI.

We aggregated the labeled ROIs into a dataset split into training and test sets, which comprised 80% (~11,000 ROIs) and 20% (~3,000 ROIs) of the data, respectively.

To further validate this AutoML approach, we also used an open dataset of ROIs imaged in the mouse primary visual cortex acquired using 2p microscopy (Stringer et al., 2019a). This ground-truth dataset contained 19,000 ROIs, from which we randomly selected 7,200 ROIs to use in the analysis to reduce compute time. We spatially and temporally downsampled the 2p ROI spatial footprint images and calcium traces, respectively, to better emulate 1p imaging data. Spatial footprints were cropped to 40 × 40 pixel images around the ROI centers, and trace data were cropped to a 500-frame time window.

Next, to generate true “negative” labels in our dataset, we manipulated 15% of ROIs (to match the proportion of positive and negative labels in our 1p dataset) using one or a combination of the following methods:

1. Modify the size of the spatial footprint by a scaling factor randomly chosen from ($\frac{1}{2}$, $\frac{1}{3}$, 2, 3; “spatial”)
2. Add gaussian noise to the calcium trace (“trace”)
3. Both methods #1 and #2 (“both”)
4. Combine two ROIs to simulate the incorrect merging of two separate ROIs detected as one ROI (“merged”)

The spatial footprints were flattened into a 1-dimensional vector and the trace data was concatenated to the end. The final 2p simulated ground truth dataset was split into training and test sets, which comprised 80% (6,000 ROIs) and 20% (1,000 ROIs) of the data, respectively.

### Model Optimization and Selection

We used two automated machine learning (AutoML) methods, TPOT (Olson et al., 2016; Olson and Moore, 2019) and AutoSklearn (Feurer et al., 2015) that are based on the popular Python machine learning toolbox, scikit-learn (Pedregosa et al., 2011) to select optimal classification models. While other AutoML tools exist that may outperform the ones we chose (Truong et al., 2019), TPOT and AutoSklearn are both free open-source, and easy to use, making them accessible for labs to incorporate into their existing analysis pipelines. To benchmark the results of the AutoML methods on the ROI curation task, we trained two types of out-of-the-box scikit-learn classifiers, Decision Tree, and K-Nearest Neighbors, with and without PCA-transformed inputs for dimensionality reduction.

The key advantage of AutoML tools such as TPOT and AutoSklearn is that they do the extensive work of finding the best type(s) of data transformation and models to build a pipeline for classifying the input data, as well as the hyperparameters associated with these steps. TPOT is an evolutionary algorithm that works with the scikit-learn API to find the best parameters and model pipelines through searching “genetic lineages” of the best performing pipelines. It will try a pipeline, evaluate its performance, and then randomly modify various parameters in search of a better pipeline (Olson et al., 2016; Olson and Moore, 2019). TPOT generates pipelines of pre-processing steps and classification models to maximize classification performance while prioritizing simpler pipelines. For example, a pipeline may consist of PCA for dimensionality reduction and a support vector machine to perform the classification, though they do not necessarily need to have multiple components. AutoSklearn performs algorithm selection and hyperparameter tuning using Bayesian optimization, meta-learning, and ensemble construction (Feurer et al., 2015) and as a result, the final classifier is an ensemble of many different model types and their associated hyperparameters. We primarily used default TPOT and AutoSklearn parameters, with a max evaluation time for a single pipeline of 10 min, and a total search time of 2 days.

During training, we used 10-fold cross-validation using stratified folds that preserved the relative proportions of “include” and “exclude” labels (i.e., during each run of training, 9 of 10 folds were used for training, and the 10th fold was used to test the performance of the model). This process was repeated for all 10 folds, resulting in an averaged performance metric for the data. We optimized the models to maximize the F1 score, the harmonic average of precision and recall, where high precision indicates a low false-positive rate and high recall indicates a low false-negative rate. In our dataset, a true positive is an extracted ROI that both the trained model and a “ground truth” human scorer define as suitable to be included for further analysis (i.e., it satisfies the three selection criteria listed above). A true negative is an extracted ROI that is excluded for further analysis by both the model and our ground truth scoring.

## Results

To define a benchmark for classifier performance on CNMF-E extracted ROIs and the utility of using trace and spatial data together, we used two conventional machine learning algorithms [Decision Trees and K-Nearest Neighbors (KNN)] trained on: (1) only spatial footprint data, (2) only trace data or (3) the combined spatial and trace data. Our spatial and trace data had a large number of features, 6,400 and 500, respectively. To test whether the classifiers would perform better using dimensionality reduction, we also compared the performance of classifiers with and without Principal Component Analysis (PCA) as a data preprocessing step. We trained and tested the Decision Tree, or the KNN classifiers on each of the three types of data listed above, with and without PCA to reduce the dimensionality of our data. The F1 scores on the test set for each of these classifiers are reported in Table 1. Notably, both the Decision Tree and K-Nearest Neighbors classifiers generalized best on the test set when trained on the combined spatial and trace data, compared to the trace only or spatial only data. The highest F1 score achieved by any of these models on our 1p ground truth dataset was the KNN (with PCA) classifier with a score of 0.885 trained on the combined data.

**Table 1:** ROI classification F1 scores in different ML and automated machine learning (AutoML) methods tested on 1p ground truth and 2p simulated ground truth data.

Classifier	1p Spatial	1p Trace	1p Combined	2p Simulated ground truth (combined)
Decision Tree	0.856	0.822	0.868	0.988
Decision Tree + PCA	0.806	0.813	0.835	0.935
K Nearest Neighbours	0.873	0.877	0.881	0.929
K Nearest Neighbours + PCA	0.814	0.815	0.885	0.953
TPOT	-	-	0.904	0.954
AutoSklearn	-	-	0.922	0.994

Next, to determine the efficacy of an AutoML approach for classification of our ROI curation task, we tested the ability of TPOT and AutoSklearn to build classifiers that can label the pre-processed spatial footprints and calcium traces of putative ROIs. Both TPOT and AutoSklearn were trained on the 11,000 labeled ROIs in the training set, split into 10 folds for cross-validation, repeated five times. The best models obtained during training were used to determine the F1 score on the test set. Table 1 reports the performance of the best models obtained by TPOT and AutoSklearn across the training folds and on the test set.

**Figure 2:**
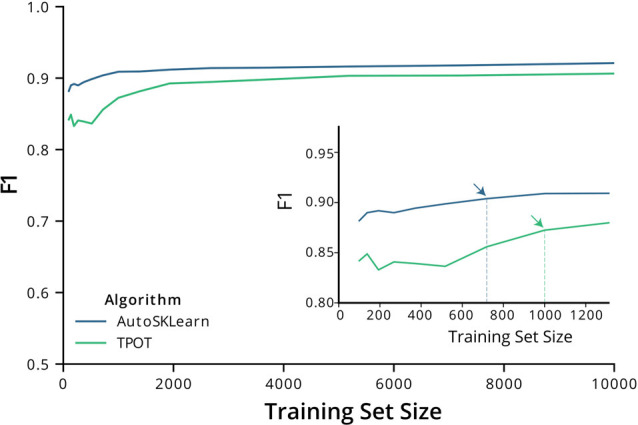
F1 score performance with increasing training size. Graph of the F1 test scores vs. the number of training samples used to train the best models output by AutoSklearn (blue) and TPOT (green). (Inset) A graph of the same plot with a smaller range of training sizes and the change point is marked with arrows on each algorithm type.

We then tested the transferability of the best classifier pipelines identified by TPOT and AutoSklearn using fewer samples. We used the top-performing classifier pipelines and hyperparameters chosen by TPOT and AutoSklearn and trained the initialized pipelines using datasets of increasing ROI numbers. The training set size ranged from 150–10,000 ROIs. Using a change point analysis algorithm (PELT, Killick et al., 2012), we determined that AutoSklean and TPOT classifiers approached a maximal F1 score with 719 and 1,000 labeled ROIs, respectively (Figure 2). The pipelines found using our much larger labeled dataset may be easily incorporated into other pipelines with a minimal computational effort to train and finetune on CNMF-E extracted ROIs from other 1p experiments, using fewer labeled ROIs.

**Figure 3:**
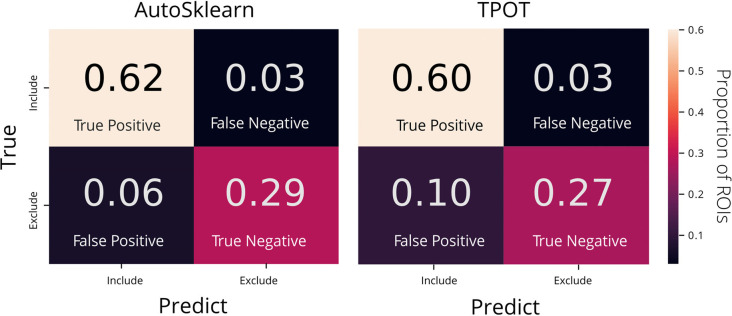
Confusion matrices of AutoML tools: TPOT and AutoSklearn. Each cell in the matrix is annotated with the proportion of ROIs labeled as Include or Exclude according to the predicted and true labels. Colors indicate the relative proportions of the labels where lower proportions are darker in color and higher proportions are lighter in color. The confusion matrices were made from predictions on the test set from the best models output by AutoSklearn (left) and TPOT (right).

To examine the classifier performance in terms of false positives and false negatives, we created confusion matrices to visualize the rate of true positives, true negatives, false positives, and false negatives from the TPOT and AutoSklearn predictions compared to the ground truth. We found that the classifier built with AutoSklearn (0.922 F1, Table 1) performs better in terms of both reducing false positives and false negatives (Figure 3).

**Figure 4:**
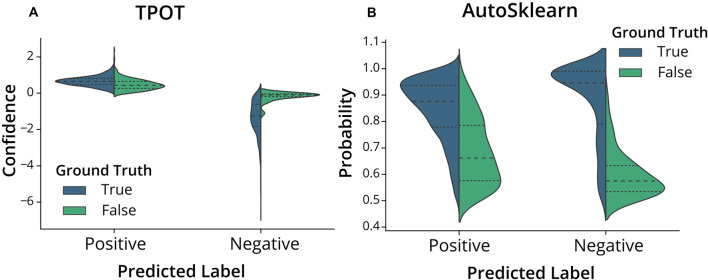
Classifier confidence (TPOT) and class probabilities (AutoSklearn) on predicted false positives and false negatives. Violin plots of the distribution of **(A)** TPOT classifier confidence or **(B)** AutoSklearn class probabilities on predicted ROI labels (Positive for Include, or Negative for Exclude) in the test set. Each half of the violin plot is the distribution of values for correct labels (True, left/blue) or incorrect (False, right/green) based on the ground truth labels.

To further assess the nature of the classification errors, we looked at the class confidences or probabilities of the test set predictions from the trained TPOT and AutoSklearn models (Figure 4). Class probabilities indicate the classifier’s certainty (using confidence score for TPOT and class probability for AutoSklearn) that a sample belongs to a particular class label. We tested whether mislabeled ROIs were also those in which the classifiers expressed less confidence in classifying. We examined the size of the difference between certainty scores (true positives vs. false positives, true negatives vs. false negatives) in TPOT and AutoSklearn using Cohen’s d (Sawilowsky, 2009; Cohen, 2013; Table 2). The AutoSklearn classifier which outperformed the TPOT classifier based on F1 scores showed large differences in certainty scores when labeling ROIs as positive (*d* = 1.36) or negative (*d* = 2.34). By contrast, the TPOT classifier was relatively less confident in both types of classification (positive *d* = 0.63, negative *d* = 1.68). In other words, the AutoSklearn classifier was more certain in applying labels to ROIs than was the TPOT classifier. This indicates that false negatives and false positives in the higher-performing AutoSklearn classifier may arise from “edge-cases” ROIs in the dataset which the classifier was not as certain about the label. In contrast, the poorer performance of the TPOT classifier may simply be due to poor generalization on the test set.

**Table 2:** Cohen’s *d* effect size of certainty scores between predicted labels that were correct or incorrect compared to ground truth in TPOT or AutoSklearn.

	Include (Positive)		Exclude (Negative)	
	TPOT	AutoSklearn	TPOT	AutoSklearn
Cohen’s *d*	0.63	1.36	1.68	2.34

**Figure 5:**
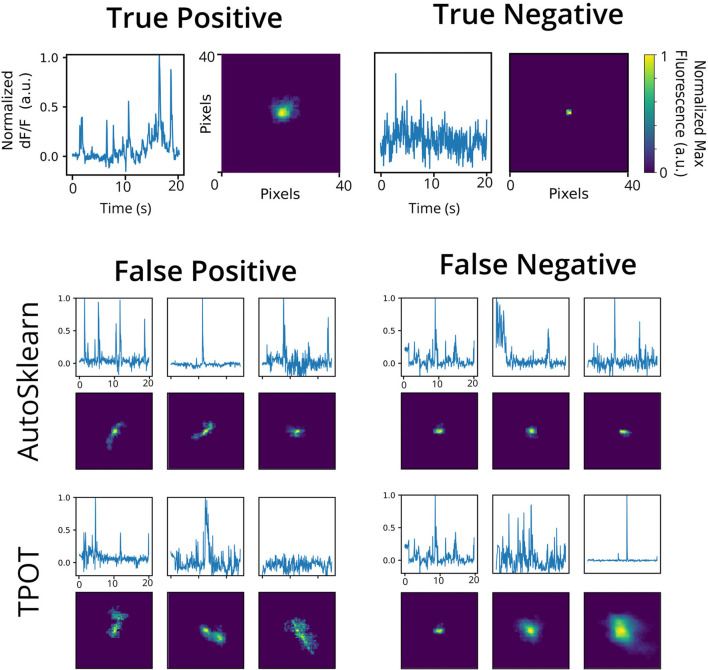
Representative false positives and negatives compared to ground truth ROIs. Example calcium traces (top) and spatial footprints (bottom) from ground truth positive- (left) and negative-labeled (right) ROIs. Example calcium traces (top) and spatial footprints (bottom) of false positive and false negative ROIs predicted from the AutoSklearn (middle row) or TPOT (bottom row) classifiers.

To investigate the nature of the false positives and false negatives from the best TPOT and AutoSklearn models, we looked at the underlying spatial footprints and calcium traces for mislabeled ROIs from both AutoML tools (Figure 5). Representative examples of excluded ROIs from the ground truth dataset show that some cells may be excluded (i.e., true negatives) because of poor trace data and/or poor spatial footprints, which possibly represent non-neuronal imaging artifacts and/or ROIs representing areas of background fluorescence. While some false positives from AutoSklearn shared similar features with true negative ROIs, others were more ambiguous. Upon inspection, these ROIs sometimes were high-quality spatial footprints with poor-quality calcium traces, or vice versa, or were composed of spatial footprints and calcium traces of true neuronal origin mixed with additional non-neuronal noise. These examples represent “edge-cases” which may be difficult to judge even by a human rater.

**Figure 6:**
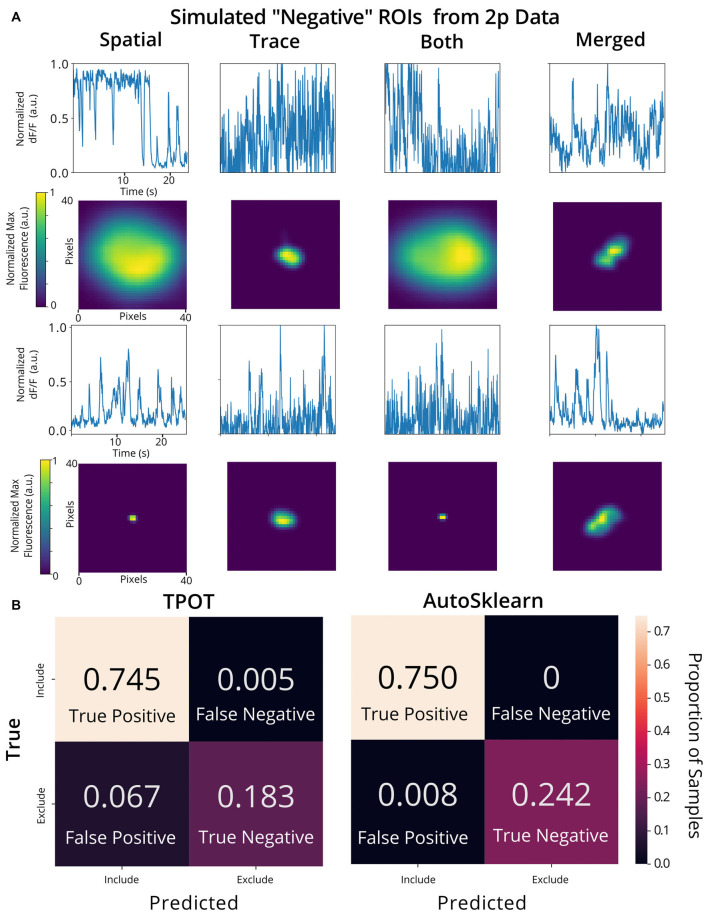
Validation of AutoML methods on modified 2p calcium imaging data. **(A)** Examples of ROIs extracted from 2p calcium imaging modified to resemble 1p data and to simulate ROIs that should be excluded from the analysis. Example trace data (top) and spatial footprints (bottom) of simulated negative ROIs that were modified using various methods: “spatial” (spatial footprints were upscaled or downscaled beyond the range of typical neuronal size in the dataset), “trace” (traces with Gaussian noise added), “both” (where both “spatial” and “trace” modifications were made) or merged” (where two ROIs are incorrectly identified as a single ROI by combining the spatial footprint and trace of two separate cells). **(B)** Confusion matrices of the TPOT (left) and AutoSklearn (right) test predictions on the 2p simulated ground truth dataset (where the test set size is 1,080 ROIs).

To validate the utility of the AutoML models on a different set of calcium imaging data, we trained and tested our out-of-the-box scikit-learn classifiers and the final ensemble classifiers output by TPOT and AutoSklearn on an open 2p calcium imaging dataset of 7,200 ROIs (a randomly chosen subset from the original 19,000 ROIs) from mouse visual cortex cells (Stringer et al., 2019a,b). We modified ROIs by downsampling the spatial footprints, and traces to simulate 1p data that has been analyzed by CNMF-E, and generated a subset of “bad” ROIs where we modified the trace, spatial footprint, both trace, and footprint modification, or simulated the merging of two separate ROIs into one ROI, a common issue in calcium imaging ROI extraction (Figure 6A). Of the conventional scikit-learn models, we found that a Decision Tree classifier achieved an F1 score of 0.988 on the 2p simulated ground truth test set (Table 1). Surprisingly, the Decision Tree classifier outperformed the TPOT Linear Support Vector Machine which had an F1 score of 0.954. However, the final AutoSklearn ensemble was achieved the best F1 score of 0.994, and notably had a far lower proportion of false positives than the TPOT classifier, and no false negatives were labeled in the test set (Figure 6B).

## Discussion

Automated curation of ROIs provides a fast, accurate method for classifying neural data generated in 1p calcium imaging experiments. We show that AutoML tools such as the open-source TPOT and AutoSklearn packages provide an easy way to build effective classifiers for ROIs extracted from the widely used CNMF-E algorithm. Spatial footprints and calcium traces from CNMF-E can be used to train these models with minimal data preprocessing. Furthermore, it may be possible to apply the top-performing classifiers generated from this work to other experimental datasets taken from different 1p imaging setups, while requiring relatively few labeled samples. Other analyses pipelines such as MIN1PIPE (Lu et al., 2018) have been developed to improve source extraction by reducing false-positive ROIs without increasing the rate of false negatives. However, given the more widespread adoption of CNMF-E, the approach described here prevents labs from having to adopt entirely new analysis pipelines. Our approach provides a balance between the need to manually review the output of CNMF-E ROIs to maximize the number of detected cells, while still allowing some further automation of the otherwise laborious curation process.

While great advancements have been made in widefield 1p imaging, fundamental constraints (e.g poor axial resolution, light scattering) limit the number of high-quality cells that can be detected compared to 2p imaging (Svoboda and Yasuda, 2006). As a result, being able to accurately detect as many “true” ROIs as possible is crucial for downstream analyses. If false positives make it through ROI extraction, or if data is lost due to false negatives, analyses of these datasets will suffer from increased variability and reduced statistical power.

AutoML may be a useful approach for curating CNMF-E extracted ROIs and can be implemented on top of pre-existing analysis pipelines without much need to adapt the software. However, there are several limitations to this approach. First, we emphasize the automated aspect of this machine learning classifier approach and little need for hand-tuning, but we recognize that the best models still make errors. Cases in which the best performing classifier generated by AutoSklearn failed to detect true positives or true negatives were further reviewed and were typically seen to be edge cases where it may be difficult for a human reviewer to make a judgment. Similarly, we found that a second expert scorer looking at the same data may not make the same judgments on such edge cases (having an interrater reliability score of 87%). While the AutoML classifiers were trained on data that had relatively little preprocessing beyond cropping and downsampling, future work could address whether feature engineering over the spatial footprints and trace data could further improve accuracy and reduce training time for model selection and hyperparameter tuning. Better curation of a training dataset for the models may help reduce ambiguous cases that make it difficult for a classifier to make accurate predictions.

Ultimately, automated methods for source extraction are still likely to be imperfect, resulting in some small proportion of false positives in the final dataset, and/or false negatives not detected during source extraction. In practice, it may be useful to be able to prioritize precision (proportion of positive labels by the classifier that are true positives), over recall (proportion of all true positives that were correctly labeled), or vice versa. For example, the loss of some small proportion of ROIs from a large dataset (i.e., false negatives) may not be as harmful as a small number of highly active false-positive ROIs that could disproportionately impact downstream analyses. We used the F1 metric, which equally weights precision and recall, to optimize our AutoML classifiers. However, one could instead use an F Beta score which is a weighted F1 score where the beta parameter determines the weight of recall (penalty on false negatives) relative to the precision (penalty on false positives). This could be used to bias the autoML search for an optimal classifier in either direction.

To reduce computing time and complexity of ROI classifiers, we flattened and concatenated our 2d spatial footprints with our 1d trace data to create a single dataset to train the classifiers on. Perhaps there are alternative machine learning methods that preserve the originally structured inputs in the spatial images and calcium trace time-series data could provide more accurate classification results. Variations on residual neural networks (ResNets) and Hierarchical Vote Collective of Transformation-based Ensembles (HIVE-COTE) have achieved a state of the art performance on image classification and time series classification, respectively (He et al., 2016; Lines et al., 2016). However, the simplicity of exported pipelines from TPOT, AutoSklearn, and conventional scikit-learn algorithms do not require as much computational power and runtime as HIVE-COTE (an ensemble of typically 37 classifiers that need to be trained and tuned for each use-case) or ResNet which typically requires a GPU to finetune the model on one’s dataset. Furthermore, the strong performance of the final extracted AutoSklearn and TPOT ensemble classifiers found using our 1p data and retrained/tested on the 2p simulated ground truth dataset demonstrates the transferable capabilities of these models with ROIs from different brain regions and different imaging set-up.

In conclusion, we present here a demonstration and benchmark of an AutoML approach for the curation of CNMF-E extracted ROIs. The data show that simple, out-of-the-box ML methods can also be trained to curate ROIs to a relatively high degree of accuracy, but the final ensemble model found by AutoSklearn was consistently able to outperform other classifiers in both the 1p and 2p simulated ground truth datasets. The methods described here can provide a flexible, free open-source, and easy-to-incorporate curation step for other researchers using CNMF-E for source extraction of their 1p datasets, while requiring few changes to their existing analysis pipelines.

## Data Availability Statement

The datasets and code generated for this study can be found in the cnmfe-reviewer GitHub repository (https://github.com/jf-lab/cnmfe-reviewer).

## Ethics Statement

The studies involving animals were reviewed and approved by Animal Care and Use Committee (ACC) of the Hospital for Sick Children. All procedures were conducted in accordance with policies of the Canadian Council on Animal Care (CCAC) and National Institutes of Health (NIH) Guidelines on Care and Use of Laboratory Animals.

## Author Contributions

LT, PF, and SJ contributed to the study design. AJ designed and constructed the CHEndoscopes. AR and AM conducted surgeries, behavior experiments, CNMF-E processing, and manual ROI labeling. LT performed analyses using automated machine learning pipelines. LT, AM, AR, and AJ performed the statistical analyses and wrote the article. All authors discussed and commented on the manuscript.

## Conflict of Interest

The authors declare that the research was conducted in the absence of any commercial or financial relationships that could be construed as a potential conflict of interest.
